# Trends in Diagnosed Posttraumatic Stress Disorder and Acute Stress Disorder in US College Students, 2017-2022

**DOI:** 10.1001/jamanetworkopen.2024.13874

**Published:** 2024-05-30

**Authors:** Yusen Zhai, Xue Du

**Affiliations:** 1 Department of Human Studies, University of Alabama at Birmingham, Birmingham; 2 Heersink School of Medicine, University of Alabama at Birmingham, Birmingham

## Abstract

This cross-sectional study investigates trends in the prevalence of diagnosed posttraumatic stress disorder (PTSD) and acute stress disorder (ASD) among US college students during a period of increased societal stresses and global health crises from 2017 to 2022.

## Introduction

Posttraumatic stress disorder (PTSD) and acute stress disorder (ASD) are critical mental health issues faced by college students, stemming from traumatic events (eg, campus shootings,^1^ sexual assault,^2^ physical violence,^2^ and natural disasters^3^). PTSD features persistent symptoms (eg, intrusion symptoms, avoidance) lasting more than 1 month after trauma, whereas ASD features similar symptoms within a 3-day to 1-month posttrauma duration. PTSD and ASD can severely impair college students’ academic and social functioning; these disorders have also been associated with long-term health issues.^2^ The broader societal implications of PTSD and ASD are profound, including reduced workforce productivity and increased health care and economic burdens.^4^ We aimed to assess trends in prevalence of diagnosed PTSD and ASD among US college students from 2017 to 2022, a period marked by escalated societal stressors and global health crises. Understanding these trends is crucial for the development of targeted, trauma-informed intervention strategies to address the urgent needs of this population during a critical developmental stage.

## Methods

This serial cross-sectional study, approved by the University of Alabama at Birmingham institutional review board, included participants from 5 waves (2017-2022) of the Healthy Minds Study across 332 US higher education institutions with diverse institutional types and geographic locations to ensure the representativeness of college student populations. All participants provided written informed consent. Detailed methods and survey designs are documented in prior publications.^5,6^ We followed the STROBE reporting guideline.

Sample weights were applied based on institutional demographics (sex, race, academic level, grade point average) to mitigate response biases. The outcome variables of this study included participants’ diagnoses of PTSD and ASD based on mental diagnoses issued by health care practitioners. Each outcome variable was dichotomized, with a value of 1 indicating a positive diagnosis for the respective condition.

Descriptive analysis calculated the weighted annual prevalence of PTSD and ASD, with the percentage change over time. Sample-weighted multivariable logistic regression estimated adjusted odds ratios (ORs) for national trends, controlling for demographic covariates (age, biological sex, race and ethnicity, international status, socioeconomic status, degree level) to reduce potential confounding effects. Two models, one for each diagnosis, assessed the change in odds of estimated prevalence from 2017 to 2022, using survey years as a continuous independent variable.^6^ Statistical significance was set at 2-sided *P* < .05. Analyses were conducted using SPSS version 28 (IBM) from January to March 2024.

## Results

Of 392 377 participants, 275 174 (57.7%, weighted) were female; 19 349 (4.9%, weighted) had diagnosed PTSD, and 1814 (0.5%, weighted) had diagnosed ASD (Table). We observed upward trends in the prevalence of PTSD and ASD from 2017 to 2022 (Figure). The prevalence of PTSD increased by 4.1 percentage points from 3.4% (2017-2018) to 7.5% (2021-2022), and that of ASD increased by 0.5 percentage points from 0.2% (2017-2018) to 0.7% (2021-2022). After adjustment for participants’ demographic differences, results from logistic regression found that the increases in the prevalence of PTSD (adjusted OR, 2.15 [95% CI, 2.06-2.24]; *P* < .001) and ASD (adjusted OR, 2.25 [95% CI, 1.96-2.58]; *P* < .001) remained statistically significant.

**Table.** Characteristics of Participating College Students, United States, 2017-2022

Characteristics	No. of participants (weighted %)				
	2017-2018	2018-2019	2019-2020	2020-2021	2021-2022
Total No.	58 681	55 393	78 283	118 590	81 430
Age, y					
18-21	33 680 (61.0)	32 684 (60.6)	44 710 (59.2)	65 832 (55.7)	47 077 (57.0)
22-25	13 768 (22.3)	11 748 (19.5)	17 303 (20.3)	25 564 (20.4)	16 579 (19.2)
26-30	6153 (8.4)	5462 (8.8)	8337 (9.7)	12 838 (9.7)	8257 (9.2)
≥31	5080 (8.4)	5499 (11.1)	7933 (10.7)	14 356 (14.2)	9517 (14.6)
Biological sex					
Female	39 738 (61.0)	37 451 (55.7)	54 139 (55.6)	85 035 (59.7)	58 811 (56.6)
Male	18 918 (38.9)	17 922 (44.3)	24 115 (44.3)	33 513 (40.3)	22 578 (43.4)
Intersex	25 (0.1)	20 (0.0)	29 (0.0)	42 (0.0)	41 (0.1)
Race and ethnicity^a^					
African American or Black	3267 (6.8)	3431 (8.7)	5259 (9.7)	12 452 (13.7)	5191 (7.1)
American Indian or Alaska Native	179 (0.5)	160 (0.3)	264 (0.3)	324 (0.4)	216 (0.3)
Asian or Asian American	9670 (13.7)	7120 (10.4)	9219 (11.2)	15 071 (9.6)	10 820 (10.4)
Latinx	4775 (8.9)	3929 (7.5)	6618 (9.8)	9194 (8.9)	9380 (13.8)
Middle Eastern or Arab American	1053 (1.7)	860 (1.5)	1266 (1.5)	1863 (1.2)	993 (1.1)
Native Hawaiian or Pacific Islander	92 (0.1)	61 (0.1)	111 (0.1)	178 (0.2)	105 (0.2)
Other (including multiracial)^b^	831 (1.5)	748 (1.7)	955 (1.5)	1310 (1.3)	905 (1.3)
White	38 814 (66.8)	39 084 (69.7)	54 591 (66.0)	78 198 (64.7)	53 820 (65.9)
Socioeconomic status (current financial stress)					
Always stressful	6930 (14.1)	6787 (13.8)	11 282 (16.0)	16 325 (15.4)	11 835 (16.7)
Often stressful	13 231 (23.9)	12 472 (23.2)	18 327 (23.7)	26 869 (23.6)	19 266 (24.0)
Sometimes stressful	21 479 (36.2)	20 137 (35.2)	27 763 (35.2)	42 038 (35.2)	28 599 (34.7)
Rarely stressful	12 735 (19.4)	12 062 (20.7)	15 870 (19.0)	24 646 (19.1)	15 877 (18.2)
Never stressful	4306 (6.5)	3935 (7.1)	5041 (6.1)	8712 (6.8)	5853 (6.5)
International status					
Domestic student	51 900 (90.0)	50 650 (92.7)	72 073 (91.7)	110 595 (94.7)	74 835 (93.1)
International student	6781 (10.0)	4743 (7.3)	6210 (8.3)	7995 (5.3)	6595 (6.9)
Degree level					
Undergraduate	41 436 (78.3)	41 789 (80.8)	58 733 (79.5)	88 267 (78.9)	61 693 (78.4)
Graduate/other	17 245 (21.7)	13 604 (19.2)	19 550 (20.5)	30 323 (21.1)	19 737 (21.6)
Diagnosed posttraumatic stress disorder	1841 (3.4)	2083 (4.1)	3579 (4.4)	6253 (5.4)	5593 (7.5)
Diagnosed acute stress disorder	149 (0.2)	217 (0.4)	365 (0.5)	531 (0.5)	552 (0.7)

^a^ Race and ethnicity were self-reported in the survey as 1 of the following: African American or Black, American Indian or Alaska Native, Asian or Asian American, Latinx, Middle Eastern or Arab American, Native Hawaiian or Pacific Islander, White, or self-identify.

^b^ Students who selected 2 or more races and/or ethnicities were classified as multiracial by the investigators and included in the other race/ethnic category along with students who selected self-identify.

**Figure.**
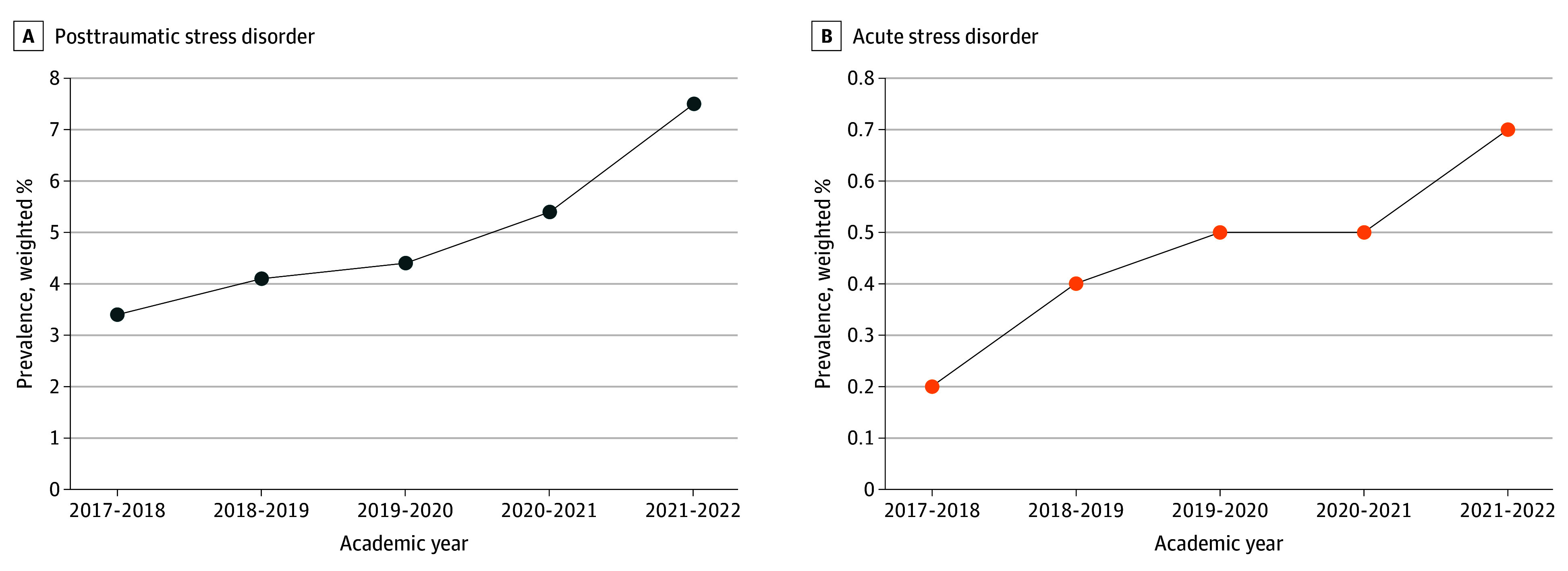
Trends in Diagnosed Posttraumatic Stress Disorder and Acute Stress Disorder Among US College Students, 2017-2022 Panel A shows the trend in weighted prevalence of diagnosed posttraumatic stress disorder. Panel B shows the trend in weighted prevalence of diagnosed acute stress disorder. Models were weighted with nonresponse weights and adjusted for age, sex, race and ethnicity, international status, socioeconomic status, and degree level.

## Discussion

In this serial cross-sectional study including a national sample of US college students, we found a notable increase in the prevalence of PTSD and ASD, rising by 4.1 percentage points and 0.5 percentage points from 2017 to 2022, respectively. These trends highlight the escalating mental health challenges among college students, which is consistent with recent research reporting a surge in psychiatric diagnoses.^6^ Factors contributing to this rise may include pandemic-related stressors (eg, loss of loved ones) and the effect of traumatic events (eg, campus shootings, racial trauma). Despite the study limitations, including the retrospective, self-reported data and single questions for diagnosed PTSD and ASD, these findings suggest the need for targeted, trauma-informed prevention and intervention strategies by mental health professionals and policy makers to support the affected student population.

## Notes

Supplement.
Data Sharing Statement
