# Supplementary material for: Trends in Diagnosed Posttraumatic Stress Disorder and Acute Stress Disorder in US College Students, 2017-2022
Source: JAMA Netw Open. 2024 May 30;7(5):e2413874. doi: 10.1001/jamanetworkopen.2024.13874 (PMC11140522; doi:10.1001/jamanetworkopen.2024.13874)
Supplement: Supplement. — Data Sharing Statement [file jamanetwopen-e2413874-s001.pdf]

Data Sharing Statement

Zhai. Trends in Diagnosed Posttraumatic Stress Disorder and Acute Stress Disorder in US College Students, 2017-2022. *JAMA Netw Open*. Published May 30, 2024. doi:10.1001/jamanetworkopen.2024.13874

Data
Data available: Yes
Data types: Deidentified participant data
How to access data: https://healthymindsnetwork.org/research/data-for-researchers/
When available: With publication

Supporting Documents
Document types: None

Additional Information
Who can access the data: researchers who propose a legitimate use of the data and are given access by the Healthy Minds Network
Types of analyses: for specific purposes
Mechanisms of data availability: with a signed data access agreement
